# High-mannose glycans from *Schistosoma mansoni* eggs are important for priming of Th2 responses via Dectin-2 and prostaglandin E2

**DOI:** 10.3389/fimmu.2024.1372927

**Published:** 2024-04-29

**Authors:** Luís Almeida, Ruthger van Roey, Thiago Andrade Patente, Frank Otto, Tom Veldhuizen, Mohan Ghorasaini, Angela van Diepen, Gabriele Schramm, Jianyang Liu, Helena Idborg, Marina Korotkova, Per-Johan Jakobsson, Martin Giera, Cornelis Hendrik Hokke, Bart Everts

**Affiliations:** ^1^Centre for Infectious Diseases, Leiden University Medical Centre, Leiden, Netherlands; ^2^Centre for Proteomics and Metabolomics, Leiden University Medical Centre, Leiden, Netherlands; ^3^Experimental Pneumology, Research Centre Borstel, Borstel, Germany; ^4^Division of Rheumatology, Department of Medicine, Karolinska Institutet, Stockholm, Sweden

**Keywords:** dendritic cells, Th2 polarization, glycans, *Schistosoma mansoni*, PGE2

## Abstract

The parasitic helminth *Schistosoma mansoni* is a potent inducer of type 2 immune responses by stimulating dendritic cells (DCs) to prime T helper 2 (Th2) responses. We previously found that *S. mansoni* soluble egg antigens (SEA) promote the synthesis of Prostaglandin E_2_ (PGE2) by DCs through ERK-dependent signaling via Dectin-1 and Dectin-2 that subsequently induces OX40L expression, licensing them for Th2 priming, yet the ligands present in SEA involved in driving this response and whether specific targeting of PGE2 synthesis by DCs could affect Th2 polarization are unknown. We here show that the ability of SEA to bind Dectin-2 and drive ERK phosphorylation, PGE2 synthesis, OX40L expression, and Th2 polarization is impaired upon cleavage of high-mannose glycans by Endoglycosidase H treatment. This identifies high-mannose glycans present on glycoproteins in SEA as important drivers of this signaling axis. Moreover, we find that OX40L expression and Th2 induction are abrogated when microsomal prostaglandin E synthase-1 (mPGES) is selectively inhibited, but not when a general COX-1/2 inhibitor is used. This shows that the *de novo* synthesis of PGE2 is vital for the Th2 priming function of SEA-stimulated DCs as well as points to the potential existence of other COX-dependent lipid mediators that antagonize PGE2-driven Th2 polarization. Lastly, specific PGE2 inhibition following immunization with *S. mansoni* eggs dampened the egg-specific Th cell response. In summary, our findings provide new insights in the molecular mechanisms underpinning Th2 induction by *S. mansoni* and identify druggable targets for potential control of helminth driven-Th2 responses.

## Introduction

Helminth parasites are known to provoke strong T helper 2 (Th2) cell-polarized immune responses, which can contribute to protective immunity but may also lead to immunopathology, yet the underlying molecular mechanisms through which helminths activate this type of immune response are still incompletely understood. A better understanding of how Th2 responses are initiated by helminths may help to identify pathways that could be targeted to shape Th2 responses in therapeutic settings, not only in the context of helminth infections but also of inflammatory disorders such as allergies that are characterized by aberrant Th2 responses.

Dendritic cells (DCs) play a highly important role in the immune system by functioning as a bridge between the innate and adaptive immune systems. Their specialized role as antigen-presenting cells (APCs) allows them to prime responses depending on the pathogen or stimulus encountered (1, 2). Upon helminth infection, DCs are key players in inducing the differentiation and activation of Th2 cells (3, 4). As such, elucidating the mechanisms by which parasite antigens license DCs to induce Th2 responses will be key to unravel how helminths activate this type of immune response.

*Schistosoma mansoni* soluble egg antigens (SEA) are one of the most commonly used antigen preparations to study the immune response against helminths (5–7). SEA is a complex mixture of highly immunogenic antigens that are capable of activating DCs, driving robust Th2-polarized immune responses (7, 8). Within SEA, omega-1 (ω1), a glycoprotein with T2 ribonuclease activity, has been identified as a key driver of Th2 responses (9), both *in vitro* and *in vivo*, by conditioning DCs for Th2 priming in a mannose receptor-dependent fashion.

Importantly, however, SEA depleted of ω1 (Δω1-SEA) can still promote a Th2 response, highlighting the existence of additional omega-1-independent mechanisms through which *S. mansoni* eggs can condition DCs for Th2 priming (5). More recently, we identified that components in SEA, independently from omega-1, can trigger a Dectin-1 and Dectin-2-dependent signaling pathway, involving Syk-dependent ERK phosphorylation, to increase COX1/2 activity, resulting in elevated oxidation of arachidonic acid and *de novo* PGE2 synthesis. This PGE2, in turn, acts in an autocrine manner on DCs to induce OX40L expression, thereby endowing DCs with the ability to prime a Th2 response (10). However, the molecular determinants in SEA that interact with Dectin-1 and Dectin-2 to induce this signaling cascade remained unidentified.

Many proteins in SEA are heavily glycosylated and their reported immunomodulatory effects are, in many cases, glycan dependent (11, 12). Correspondingly, on DCs, SEA has been shown to interact with and signal through several glycan-binding C-type lectin receptors, such as the macrophage galactose-type lectin (MGL), CD209 (DC-SIGN), and CD206 (mannose receptor) to modulate TLR-induced cytokine production and T cell-priming capacity (13–17). The main glycan moieties present in SEA that are thought to mediate interaction with these receptors are Galβ1,4(Fucα1,3)GlcNAc (LeX), GalNAcβ1,4(Fucα1,3)GlcNAc (LDNF), and GalNAcβ1,4GlcNAc (LDN) (13, 18). However, it is currently unknown which component(s) in SEA act as ligands for Dectin-1 and Dectin-2. Both transmembrane C-type lectins are well known to bind β-Glucan and high-mannose glycans, respectively (19, 20). As SEA has been shown to contain high-mannose glycans (21) and since Δω1-SEA requires Dectin signaling (10), we here explored whether SEA components were capable of binding to and, subsequently, activating signaling downstream of Dectin-1- and Dectin-2.

Additionally, it remains to be determined whether pharmacological targeting of the PGE2/OX40L signaling pathway in DCs, in particular PGE2 synthesis, could be used to manipulate the egg-induced Th2 response. We previously found that while antibody-mediated PGE2 neutralization was able to fully block omega-1 independent Th2 priming by DCs, COX1/2 inhibition only had a partial effect (10). This prompted us to explore the possibility that a more targeted pharmacological intervention is needed, i.e., by selectively inhibiting PGE2 synthesis to effectively block Th2 priming.

## Materials and methods

### Preparation and purification of *S. mansoni* egg-derived antigens

SEA and Δω1-SEA from *S*. *mansoni* eggs were prepared and isolated as described previously (22).

### EndoH treatment of SEA and Δω1-SEA

SEA and Δω1-SEA were treated with Endo-H (NEB #P0702S) according to their non-denaturing protocol conditions. Succinctly, for both SEA and Δω1-SEA, 500 µg was treated with Endo-H and 500 µg was mock-treated. The samples were not denatured. Buffer was directly added along with 12.500 units of Endo-H. The samples were then incubated at 37°C for 24 h. Removal of oligomannose N-glycans was confirmed by mass spectrometry (**Supplementary Figure S1**).

### Purification of mannose-9 from human serum

High-mannose glycans were cleaved from human serum proteins using Endo-H (NEB #P0702S) and incubated at 37°C for 48 h. The released glycans were purified by the application of C18 SPE (J.T.Baker, #7020-03) and carbon SPE columns (Supelco, #57088) and labeled with anthranilic acid (AA) by reductive amination. To remove the excess labeling reagent, acetonitrile (ACN) was added to a final concentration of 75%, and the sample was loaded onto Bio-Gel P10 Gel resin (catalog no.: 1504144; Bio-Rad) previously conditioned with 80% ACN. The glycans were eluted with MQ and dried using Speedvac. The glycans were then further fractionated using reverse-phase HPLC (RP-HPLC) and analyzed by MALDI-TOF MS, yielding pure Man9 glycan. The above-mentioned methodology is described in more detail by Petralia et al. (23)

### AA to AEAB label conversion

2-Amino-N-(2-aminoethyl)-benzamide (AEAB)-labeled Man9 glycans were generated from AA-labeled Man9 as previously described (24). In short, 20 µg of both Maltopentose-AA [generated from maltopentose (Sigma #SMB01321) as described for Man9 above] and Man9-AA were mixed with 50 µL EDC (10 mg/mL in DMSO) and 50 µL HOBt (10mg/mL in DMSO). Subsequently, we added 10 µL of 5% (v/v) EDA and 0.5 M MES buffer (pH = 6.5). The samples were then vortexed for 1 min and incubated at room temperature for 3 h and then quenched with 1.1 mL of cold ACN. The samples were then vortexed and stored at -20°C for 30 min. The cloudy reaction mixture was centrifuged for 10 min at 10,000 × *g*. The supernatant was discarded and the precipitate was dried under vacuum. Once dry, it was dissolved in 100 µL of Mili-Q and applied to a RP-HPLC C18 column for purification.

### Generation of Man9-labeled NHS gold nanoparticles

Both Man9-AEAB and Maltopentose-AEAB were conjugated to 100 nm NHS-activated gold nanoparticles using a kit (Cytodiagnostics #CGN10K-100-1) and following the manufacturer’s protocol. As a deviation to the protocol, the glycans were diluted to 0.1 μg/μL using protein re-suspension buffer, and 1× PBS was used instead of conjugate storage buffer. The reaction efficiency was estimated to be ≈58% via RP-HPLC with fluorescence detection to quantify the percentage of recovered uncoupled material.

### Human DC culture, stimulation, and analysis

Peripheral blood mononuclear cells were isolated from the venous blood of healthy volunteers by density centrifugation in Ficoll as described before (25). Monocytes were isolated by positive magnetic cell sorting using CD14-microbeads (Miltenyi Biotech, Bergisch Gladbach, Germany) and cultured in 10% FCS RPMI medium supplemented with 20 ng/mL rGM-CSF (BioSource/Invitrogen, Carlsbad, CA, USA) and 0.86 ng/mL of rIL-4 (R&D Systems, Minneapolis, MN, USA). On days 2 and 3, the medium, including the supplements, was replaced.

Immature moDCs were stimulated on days 5 and 6 in the presence or absence (if indicated) of 25 ng/mL ultrapure LPS (*Escherichia coli* 0111 B4 strain; InvivoGen, San Diego, CA, USA) along with the indicated reagents: SEA (20 μg/mL), Δω1-SEA (20 μg/mL), 50 μg/mL Zymosan (Z4250; Sigma-Aldrich, St. Louis, MO, USA), CIII (10 µM), indomethacin (Sigma-Aldrich #I7378, 50 µM), and 5, 2, or 0.2 μg/mL of either Man9- or Maltopentose-bound nanoparticles or inactivated nanoparticles.

After 48 h of stimulation, the surface expression of co-stimulatory molecules was determined by flow cytometry (FACS-Canto; BD Biosciences, Breda, The Netherlands or Aurora; Cytek, Amsterdam, The Netherlands) using the following antibodies: CD1a (clone HI149), CD14 (clone MΦP9), CD86 (clone 2331 FUN-1), CD40 (clone 5C3), and CD80 (clone L307.4) (all BD Biosciences); HLA-DR (clone LN3) CD83 (clone HB15e) (both eBioscience, San Diego, CA, USA); and CD252/OX40L (clone ANC10G1; Ancell, Bayport, MN, USA). Only live cells that were negative for Zombie NIR (BioLegend Europe BV, Amsterdam, The Netherlands) were included in the analysis. The acquired samples were unmixed using SpectroFlo version 3 (if measured with Aurora) and analyzed with FlowJo.

### Human DC and T cell coculture and determination of T cell polarization

For the analysis of T cell polarization, 5 × 10^3^ moDCs pulsed for 48 h were cultured with 2 × 10^4^ allogenic naïve CD4+ T cells for 7 days in the presence of staphylococcal enterotoxin B (10 pg/mL). On day 7, the T cells were replated and rhuIL-2 (10 U/mL; R&D Systems) was added to expand the T cells. On days 9 and 10, the T cells were split with medium containing the same concentration of rhuIL-2. Intracellular cytokine production was analyzed on day 12 after restimulation with 100 ng/mL phorbol myristate acetate, 2 μg/mL ionomycin, and 10 μg/mL brefeldin A for 4 h. Subsequently, the cells were fixed with 2% paraformaldehyde (all Sigma-Aldrich). The cells were permeabilized with permeabilization buffer (eBioscience #00-5523-00) and stained with antibodies against IL-4 and IFN-γ, respectively (BD Biosciences). The acquired samples were unmixed using SpectroFlo version 3 (if measured with Aurora) and analyzed with FlowJo.

### Measurements of PGE2 levels in culture supernatants

Lipid mediators (LM) and polyunsaturated fatty acids (PUFA) were measured using reverse-phase liquid chromatography coupled to tandem mass spectrometry (RPLC-MS/MS) as previously described (26), with some modifications. Briefly, 2 µL internal standard (IS) mix of deuterated lipid standards consisting of PGE2-d_4_, 15-HETE-d_8_, Leukotriene B_4_-d_4_, DHA-d_5_, 8-iso-PGF2a-d_4_, and 14 (15)-EET-d_11_ (50 ng/mL in MeOH) was added to 400 µL culture supernatants. Lipids were extracted and purified by solid-phase extraction (SPE) after protein precipitation with 1.2 mL MeOH. The dried extracts were reconstituted in 100 µL 40% MeOH and transferred into a micro-vial glass insert. Furthermore, a 40-µL sample was injected and analyzed using a Shimadzu Nexera LC40 system with an autosampler coupled to a QTrap 6500 mass spectrometer (Sciex). Kinetex C18 50 × 2.1 mm, 1.7 µm column, and C8 precolumn (Phenomenex) were used for LC separation. LC–MS/MS chromatograms were integrated manually using Sciex OS (Sciex). The results were reported as relative peak area of lipids to the internal standards. PGE2-d_4_ IS was used for reporting the area ratios of PGE2, TxB2, and PGF2a.

### ERK phosphorylation

For the detection of ERK phosphorylation (pERK), 2.5 × 10^4^ immature moDCs were seeded overnight in a 96-well flat-bottom plate. moDCs were stimulated with SEA (25 μg/mL) and Δω1-SEA (25 μg/mL) for the indicated periods, and the moDCs were fixed for 15 min with 4% ultrapure formaldehyde (Polysciences, Warrington, PA, USA) directly in the plate. The cells were harvested and washed first with PBS and then with 0.5% of saponin for permeabilization. The cells were intracellularly stained with anti-phospo-p44/42 MAPK (Erk1/2) (clone E10) (both Cell Signalling Technology). Following 2 h of incubation at room temperature, the cells were washed with 0.5% of saponin, and ERK phosphorylation was determined by flow cytometry.

### Dectin ELISA

For the Dectin-1/2-hFc binding ELISAs, 96-well high-binding half-area microplates (Corning #10052511) were used. The appropriate antigens were coated in 50 μL TSM (20 mM Tris-HCl, 150 mM NaCl, 2 mM CaCl_2_, and 2 mM MgCl_2_ at pH 7.4) overnight at 4°C; SEA of *Schistosoma mansoni* (50 μg/mL), mock or Endo-H treated SEA of *Schistosoma mansoni* (50 μg/mL), Zymosan (20 μg/mL), NHS gold nanoparticles (5 μg/mL), or 1% BSA in TSM. After overnight coating, the plate was washed three times with an excessive amount of TSM/0.005% Tween. After washing, the plate was blocked for 1 h with 100 μL TSM/1% BSA at room temperature. After blocking, the plate was washed three times, again with an excessive amount of TSM/0.005% Tween. Following the washing, 50 μL Dectin-1-hFc (Sino Biological #10215-H01H) or Dectin-2-hFc (Sino Biological #10250-H01H) at a concentration of 10 μg/mL in TSM/0.005% Tween was incubated for 2 h at room temperature. After incubation, the plate was washed five times again with an excessive amount of TSM/0.005% Tween. Next, 50 μL of Monoclonal Biotin Mouse anti-human IgG1-Fc (Invitrogen #05-3340) (1:500 in TSM/0.005% Tween) was added to the plates, along with HRP-Strep (BD 51-9002813), and incubated for 1 h. The plate was then washed again for six times. We used 50 μL of TMB ELISA substrate solution (ThermoFisher #34021) for 30 min, followed by 25 μL of H_2_SO_4_ 1.8 M to stop the coloring reaction. ELISA readout was performed at 450 nm, with absorbance correction at 570 nm, using MultiskanTM FC Microplate Photometer (ThermoFisher, type 357).

### Mice

Wild-type (WT) mice, both male and female and all on a C57BL/6J background, were bred under SPF conditions at the Leiden University Medical Center (LUMC), Leiden, The Netherlands. The mice were culled through cervical dislocation. Animal experiments were performed when the mice were between 8 and 16 weeks old. The animal experiments were performed in accordance with local government regulations, EU Directive 2010/63EU, and Recommendation 2007/526/EC regarding the protection of animals used for experimental and other scientific purposes as well as approved by the Dutch Central Authority for Scientific Procedures on Animals (CCD) (animal license number AVD116002015253).

### SEA immunization

The mice were injected subcutaneously with 5,000 *S. mansoni* eggs in the hind footpad and injected i.p. with either vehicle or 1 mg CIII/20 g of body weight. The injections with either CIII or vehicle were then repeated on days 2 and 4 post-immunization. At 7 days later, the mice were sacrificed, and cells from both draining and nondraining lymph nodes were isolated and analyzed as described below.

### Analysis of murine T cell responses

Antigen-specific responses were determined by culturing 5 × 10^5^ LN cells per well in high-binding 96-well flat-bottom plates (Corning #3590) in 200 μL complete medium (RPMI containing 10% FCS, 100 U/mL penicillin/streptomycin, and 2 mM L-glutamine) in the presence of 20 μg/mL SEA along with 2.5 μg/mL IL-4R blocking antibody to retain IL-4 in culture supernatants. After 48 h, the culture supernatants were stored for cytokine determination. The cell culture supernatants were analyzed for cytokines using the Cytokine Bead Array (BD) according to the manufacturer’s recommendation. The samples were analyzed on BD Canto II Flow Cytometer. Alternatively, cytokine production was assessed by intracellular staining of T cells from LNs after polyclonal restimulation in 96-well flat-bottom plates for 4 h with phorbol 12-myristate 13-acetate (PMA; 50 ng/mL), ionomycin (1 μg/mL), and brefeldin A (10 μg/mL; all from Sigma-Aldrich). Afterward, the cells were fixed with 2% PFA and subsequently stained in eBioscience permeabilization buffer: IL-4 (11B11), IFN-γ (XMG1.2), IL-13 (eBio13A), IL-17A (TC11-18H10.1), and CD4 (RM4-5), IL-10 (JES5-16E3) (all BD Bioscience or BioLegend). The samples were analyzed on BD Canto II Flow Cytometer or Cytek Aurora. The acquired samples were unmixed using SpectroFlo version 3 (if measured with Aurora) and analyzed with FlowJo.

### Statistical analysis

Data were tested for normality using the Shapiro–Wilk test. The statistical tests used are indicated in the figure legends. Generally, data were compared using one-way ANOVA for more than two groups or two-way ANOVA for comparing multiple parameters across two or more groups, with Tukey’s *post-hoc* test for multiple comparison. If comparing parameters within the same sample, a paired or repeated-measures test with Geisser–Greenhouse correction was used. *p*-values <0.05 were considered significant (*p < 0.05, **p < 0.01, ***p < 0.001, ****p < 0.0001). All statistical analyses were performed using GraphPad Prism v.9.0.

## Results

### SEA binds to Dectin-2 in a high-mannose-dependent manner

**Figure 1:**
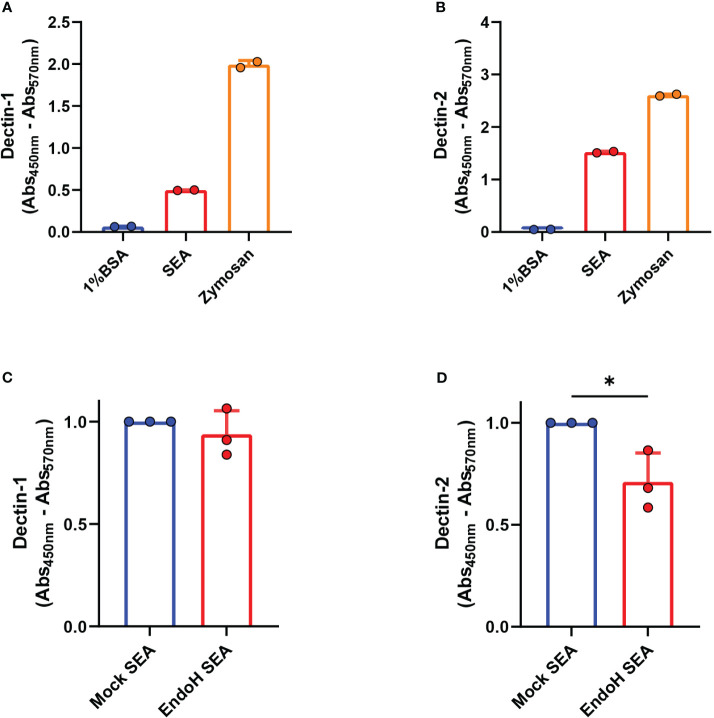
Dectin-1 and Dectin-2 directly bind to soluble egg antigens (SEA). Binding ELISA of the indicated molecules was performed as described in “Materials and methods” for **(A)** Dectin-1 and **(B)** Dectin-2. Binding ELISA of the indicated molecules for **(C)** Dectin-1 and **(D)** Dectin-2. **(A, B)** Representative plots of three experiments (*n* = 2 per experiment, mean ± SD). **(C, D)** Pooled data of three independent experiments shown with data normalized by the mock SEA condition and compared using a paired one-tailed *t*-test (*n* = 2 to 3 per experiment, mean ± SD). *p < 0.05.

We previously reported that blocking antibodies against Dectin-1 and Dectin-2 in DCs were able to impair omega-1-independent Th2 priming by SEA (10). However, it was not assessed whether the components in SEA were directly interacting to Dectin-1 and/or Dectin-2 to promote this response. To determine this directly, we performed a binding ELISA with a construct consisting of the carbohydrate binding domain of Dectin-1 or Dectin-2 coupled to a human IgG1 Fc domain. Zymosan, a known ligand for Dectin-1 and Dectin-2, was taken along as positive control. We observed that both Dectin-1 and Dectin-2 were directly able to bind to the components present in SEA (**Figures 1A, B**).

These receptors are C-type lectins that preferentially bind to sugar residues. It was shown in previous studies (8) that high-mannose glycans (e.g., Man9), known to be Dectin-2 ligands (20, 27), are present in high frequency in the mixture. To assess if the presence of these high-mannose moieties was required for SEA to interact with Dectin-1 and Dectin-2, we treated SEA with endoglycosidase-H (EndoH) to specifically remove N-linked oligomannose glycans, including those with high mannose, without affecting complex N-linked glycans or O-linked glycans (**Supplementary Figure S1**). EndoH hydrolyzes the glycosidic linkage of high-mannose glycans between GlcNAc1 and GlcNAc2, resulting in free glycans with only one GlcNAc residue present in the core, while PNGase-A cleaves off glycans between GlcNAc 1 and asparagine, resulting in free glycans with two GlcNAc residues present in the core. The success of EndoH treatment can thus be confirmed in the top spectrum due to the absence of high-mannose glycans with two GlcNAc residues, indicating that all high-mannose glycans were released by Endo-H before the PNGase-A treatment.

As assessed in a Dectin-1 and Dectin-2 binding ELISA, the hydrolysis of high-mannose glycans in SEA did not affect binding by Dectin-1. However, this treatment did significantly decrease the binding of SEA by Dectin-2, suggesting that SEA directly interacts with the latter in a partly high-mannose-dependent manner (**Figures 1C, D**).

### High-mannose glycans in SEA are required, but not sufficient, to induce PGE2 and OX40L expression and Th2 priming by moDCs

**Figure 2:**
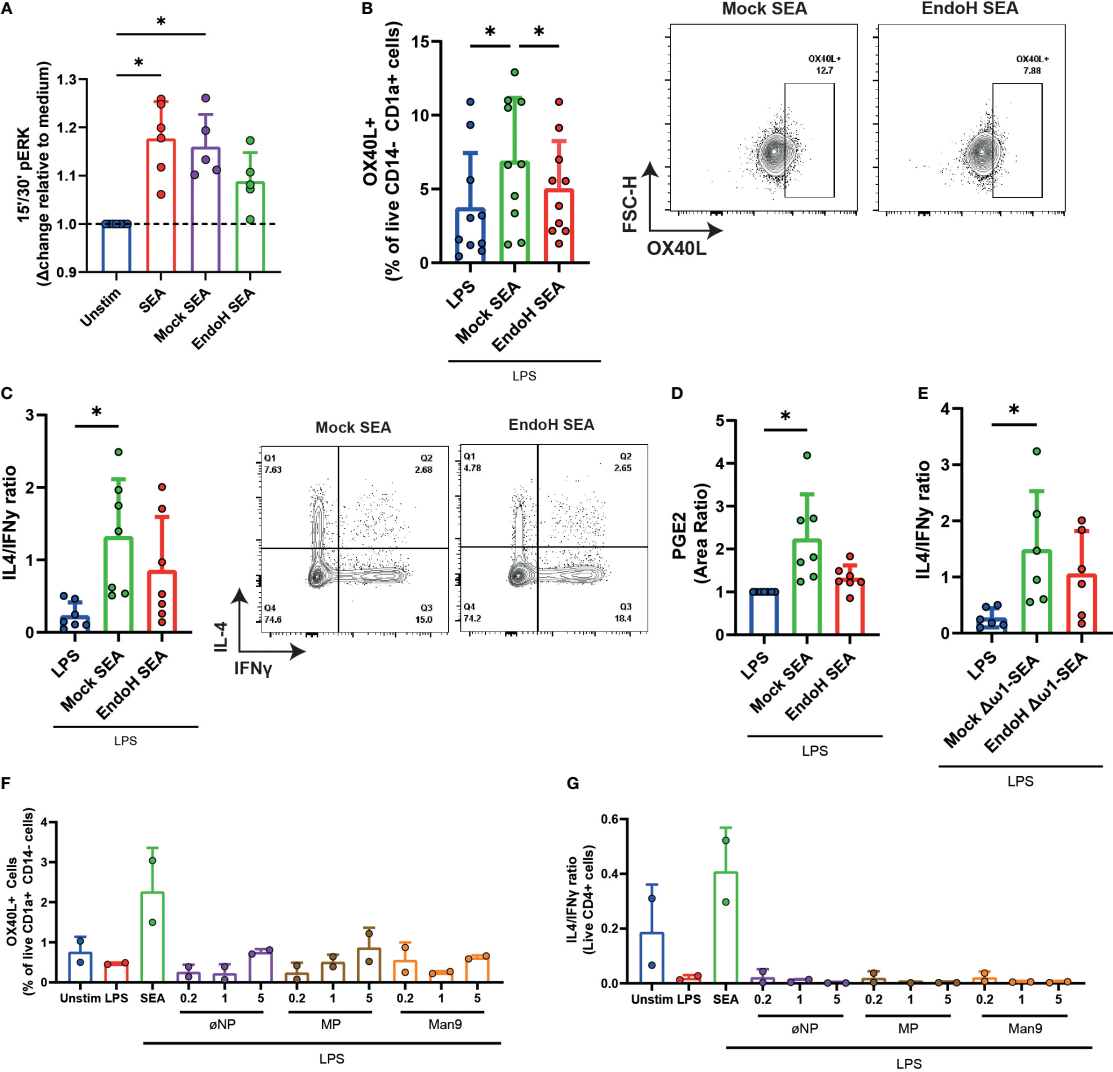
Man9 is required, but not sufficient, to induce OX40L in moDCs and subsequent Th2 priming. **(A)** ERK phosphorylation was measured with FACS after stimulating moDCs for 15 and 30 min with the indicated stimuli. **(B)** OX40L expression by moDCs was measured via FACS after 48 h of stimulation with mock-treated SEA or with EndoH-treated SEA, all in the presence of lipopolysaccharide. **(C)** Th1 and Th2 priming abilities of moDCs treated with the indicated stimuli were analyzed as described in “Materials and methods”. The ratios of IL4+IFNy- percentage over IL4-IFNy+ percentages are based on intracellular staining following PMA/Iono/BrefA stimulation. **(D)** PGE2 levels (measured in area ratio) in supernatants from moDC cultures after stimulation with the indicated stimuli. **(E)** IL4/IFNy ratio of CD4+ T cells primed by moDCs treated for 48 h with the indicated stimuli. **(F)** moDCs were stimulated with inactivated nanoparticles (øNP) Maltopentose-coated nanoparticles (MP) or Man9-coated nanoparticles, and OX40L expression was measured via FACS. **(G)** Th1 and Th2 priming abilities of moDCs treated with the indicated nanoparticles were also measured, as mentioned in **(C, E)**. **(A–D)** Data points represent individual donors pooled from 3–5 experiments with data compared using a paired one-way ANOVA (*n* = 5–10, mean ± SD). **(F, G)** Data points represent data from two individual donors. *p-value <0.05.

Considering that SEA requires the presence of oligomannose glycans, including Man9, to bind to Dectin-2, we wondered if the presence of these glycans was important for the induction of the signaling cascade leading to OX40L expression and Th2 polarization by moDCs. EndoH-treated SEA lost its ability to induce ERK phosphorylation (**Figure 2A**), OX40L expression (**Figure 2B**), and subsequent Th2 polarization by moDCs (**Figure 2C**). The latter two readouts were performed in the presence of LPS as neutral DC maturation factor (5). Correspondingly, the synthesis of PGE2 was also reduced in moDCs stimulated with EndoH-treated SEA (**Figure 2D**). These effects were not confounded by the presence of omega-1 (ω1) as moDCs stimulated with SEA depleted of ω1 (Δω1-SEA), which had undergone EndoH treatment, were also compromised in their ability to induce a Th2 response compared to DCs stimulated with mock-treated control Δω1-SEA (**Figure 2E**). These data suggest that high-mannose residues are important for ω1-independent Th2 polarization by SEA.

To investigate if high-mannose glycans themselves are sufficient to recapitulate the effects of SEA, we coupled AEAB-labeled mannose-9 (Man9) oligosaccharides isolated from human serum to N-hydroxysuccinimide (NHS)-activated gold nanoparticles. Interestingly, while these loaded nanoparticles were efficiently covered with high-mannose glycans (**Supplementary Figures S2A–D**) and were able to directly bind to Dectin-1 and Dectin-2 as determined by ELISA (**Supplementary Figures S3A, B**), they were not able to replicate the effects of SEA on moDCs in terms of inducing OX40L on moDCs or condition them for Th2 polarization in a concentration of glycans labeled to NPs ranging from 0.2 to 5 µg/mL (**Figures 2F, G**). This indicates that, while high-mannose glycans are required for the activation of the dectin-OX40L axis by SEA, they are not sufficient to activate this pathway.

### Selective inhibition of PGE2 synthesis impairs OX40L expression and Th2 priming by moDCs

We previously found that SEA-driven Th2 polarization via Dectin-1/2 is critically dependent on PGE2, one of the downstream products of COX. However, in contrast to PGE2 neutralization experiments, COX inhibition was only able to modestly decrease Th2 priming (10). We wondered whether this could be explained by the fact that COX inhibition does not only inhibit PGE2 synthesis but also affects the synthesis of other COX products that may affect the Th2 priming capacity of DCs. To test this and avoid this potentially confounding issue, we used CIII, an inhibitor that specifically targets microsomal Prostaglandin E synthase-1 (mPGES) without affecting other COX-derived products (28) (**Supplementary Figure S4**).

**Figure 3:**
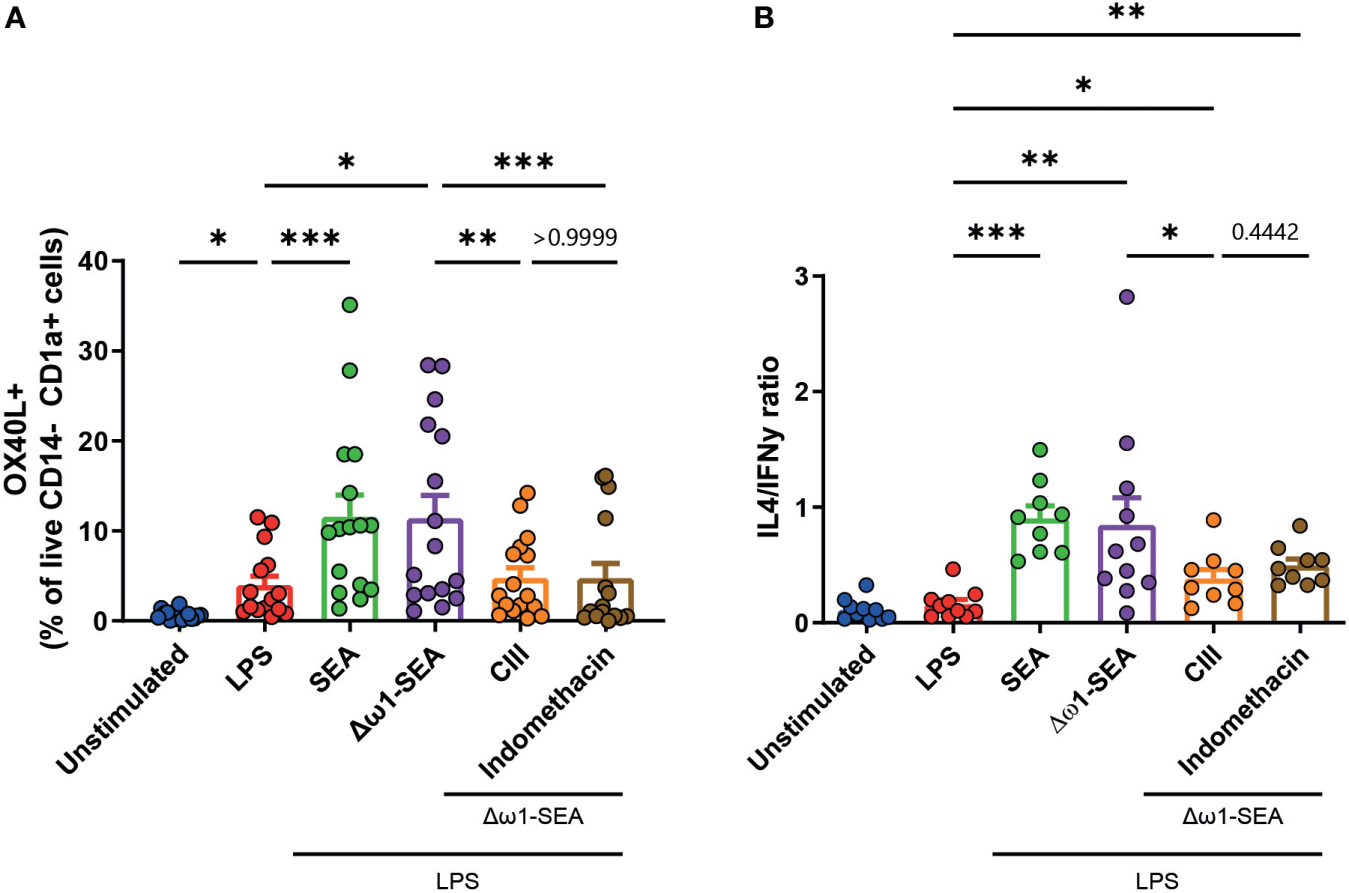
Selective inhibition of mPGES, but not general COX inhibition, in moDCs reduces Th2 priming following soluble egg antigen stimulation. **(A)** OX40L expression in moDCs following 48 h of stimulation with the indicated stimuli and **(B)** Th1 and Th2 priming abilities as described in **Figure 2**. Data points in **(A, B)** represent data from seven to 16 individual donors. Data were compared using a paired one-way ANOVA (median ± SD). *p < 0.05, **p < 0.01 ***p < 0.001.

Corresponding with the inhibition of PGE2 synthesis by both drugs, their incubation reduced the Δω1-SEA-driven expression of OX40L by moDCs (**Figure 3A**). While no statistical difference was found when comparing CIII to indomethacin (*p* = 0.442) (**Figure 3B**), CIII treatment significantly lowered the Th2 priming ability of Δω1-SEA-treated moDCs. In contrast, this was not the case for treatment with indomethacin, suggesting that targeting PGE2 synthesis itself is superior in modulating Th2 priming in this setting to targeting COX further upstream.

### Inhibition of PGE2 synthesis impairs the T cell response to *S. mansoni* eggs *in vivo*

In view of these results that we obtained *in vitro*, we wondered if PGE2 synthesis inhibition would also reduce Th2 polarization *in vivo*. To test this, we injected *S. mansoni* eggs in the footpad of wild-type mice, followed by i.p. injections of CIII on days 0, 2, and 4 post-challenge. On day 7, the mice were sacrificed, and the CD4^+^ T cell response in the draining and non-draining lymph nodes was characterized.

**Figure 4:**
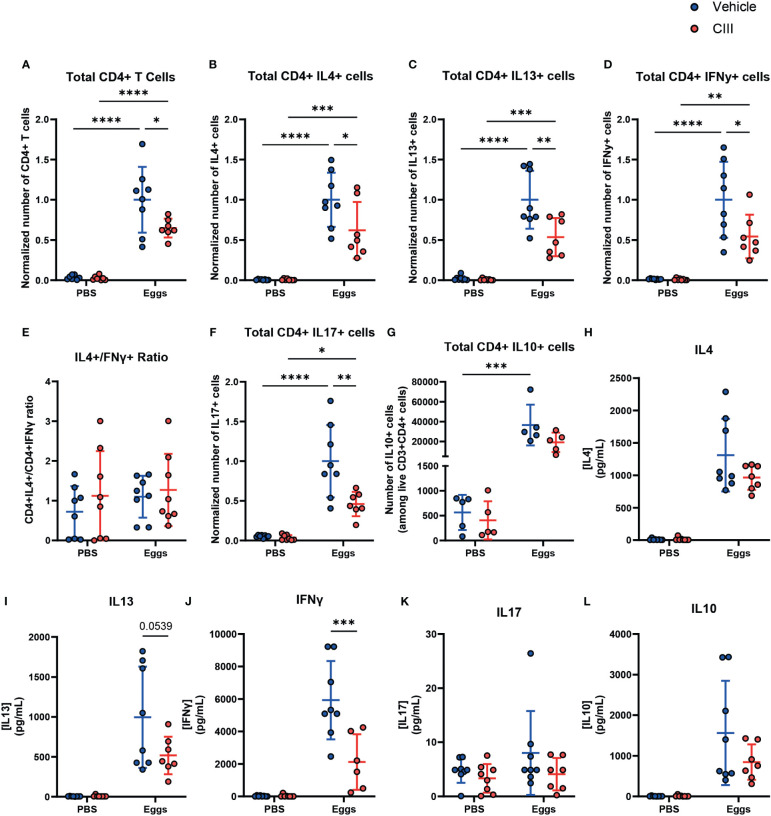
*De novo* PGE2 synthesis is required for the optimal induction of CD4^+^ T cell responses by *S. mansoni* eggs *in vivo*. **(A)** Total number of live CD4^+^ T cells in non-draining and draining lymph nodes in mice injected with CIII or vehicle following injection with *S. mansoni* eggs and total number of live CD4^+^ T cells producing IL4 **(B)**, IL13 **(C)**, and IFNy **(D)**, along with the ratio of IL4+/IFNy+ **(E)**, are shown. CD4+ T cells producing IL17 **(F)** and IL10 **(G)** were also measured. **(H–L)** Antigen-specific cytokine production of the indicated *ex vivo* T cells was measured using a CBA assay in supernatant collected after 24 h of stimulation with soluble egg antigen. Data points represent individual mice from two experiments. Data were compared using a two-way ANOVA (*n* = 7 to 8, mean ± SD). The number of cells was normalized by using the average of vehicle + eggs condition from each respective experiment. *p < 0.05, **p < 0.01, ***p < 0.001, ****p < 0.0001.

Mice challenged with *S. mansoni* eggs and treated with CIII displayed an overall lower number of CD4^+^ T cells in draining LNs (**Figure 4A**), resulting in a lower number of IL4- and IL13-producing Th2-polarized T cells upon polyclonal restimulation, when compared to egg-immunized mice injected with the vehicle control. However, this was also true for the number of IFNγ- and IL-17-producing Th cells. As a consequence, CIII treatment did not alter the ratio between IL4- and IFNγ-producing CD4^+^ T cells (**Figures 4B–F**). No difference was seen in IL10-producing CD4^+^ T cells (**Figure 4G**). To assess antigen-specific cytokine responses, cells that had been isolated from the LNs of immunized mice were restimulated with SEA. While no difference was seen in the IL-4 response (**Figure 4H**), cell cultures from egg-immunized mice receiving CIII displayed a nearly significant lower IL-13 (**Figure 4I**) and a decreased IFNγ (**Figure 4J**) response to the antigens, while the IL-17 (**Figure 4K**) and the IL-10 (**Figure 4L**) levels remained unaffected. This indicates that the optimal priming of Th2 cell responses upon *S. mansoni* egg challenge *in vivo*, as well as that of other concomitant egg-induced Th cell responses, relies on *de novo* PGE2 synthesis.

## Discussion

It has been previously shown that SEA drives type 2 immune responses, even if depleted of ω-1, one of its main Th2-inducing molecules (5, 10). While it was demonstrated that this ω-1-independent Th2 priming was reliant on dectin signaling and PGE2 synthesis in DCs, the ligands in SEA that trigger this signaling cascade had not been identified, nor had it been determined if the selective chemical inhibition of PGE2 synthesis in DCs would impair their Th2 priming abilities following SEA stimulation.

Here we have shown that Dectin-1 and Dectin-2 are able to directly bind components in SEA and that for Dectin-2 this is, in part, dependent on the presence of high-mannose N-glycans. Dectins are traditionally associated with anti-fungal responses (29–31); however, we show that both Dectin-1 and Dectin-2 can also bind to helminth-derived glycoproteins. This aligns with previous work showing that both Dectin-1 and Dectin-2 play a role in driving the immune response against other helminths (32, 33).

While Dectin-2 is known to bind to mannose residues (20, 27), we have demonstrated that Dectin-1, which typically binds to β-Glucans (34), can also bind to SEA, albeit in a high-mannose-independent manner. The motifs in SEA behind Dectin-1 binding remain elusive, as SEA does not contain β-glucans typically found in fungi (21). However, there is data showing that Dectin-1 can bind to N-glycans present on tumor cells (35) and to an unknown ligand present in T cells that was resistant to tunicamycin/N-glycosidase treatment but susceptible to trypsin treatment (36), suggesting that Dectin-1 may bind to other carbohydrates than classically thought and perhaps even non-glycan components.

The importance of high-mannose glycans present in SEA in mediating binding to Dectin-2 was further extended in functional studies in which treatment of SEA with the enzyme EndoH, to hydrolyze high-mannose glycans from its glycoproteins, not only reduced Dectin-2 binding but also translated into lower pERK levels, PGE2 synthesis, and OX40L expression and, subsequently, impaired Th2 priming ability by moDCs. However, while we here provide evidence for a requirement of high-mannose glycans in promoting this Dectin-2/PGE2/OX40L signaling axis by SEA, these glycan moieties alone do not appear to be sufficient to drive this response, as loading gold nanoparticles with Man9 residues did not mimic the effects seen with SEA. This might be due to possible differences in the coating density of Man9 glycans between the native proteins and the nanoparticles (37), as glycan density may influence the extent to which multimers of Dectin-2 (or other glycan-binding receptors) can be formed, which can affect the signaling strength downstream of those receptors (38). Alternatively, there might be a contribution of an unknown co-receptor engaged by other glycans or proteins in SEA to the signaling cascade—for instance, we previously reported that CD206 on moDCs is needed for the optimal expression of PGE2 following SEA stimulation (10), which may suggest that Dectin-2 may act in concert with other glycan-binding receptors to drive this response. Finally, a not mutually exclusive possibility is that Man9 facilitates Dectin-2-dependent endocytosis of a carrier protein that subsequently modulates DC function for Th2 priming, analogous to what had been previously described for ω-1, that requires its glycans to be internalized after which its ribonuclease activity modulates DC function (9).

In addition, we demonstrate that selective chemical inhibition of PGE2 synthesis is superior in inhibiting Th2 polarization by DCs stimulated with helminth antigens compared to targeting COX. Some conflicting results have been published regarding the effects of COX2 inhibition on Th1 and Th2 responses. While some studies indicate that COX2 activity/PGE2 synthesis induces a Th2 response (39–42), some point to the opposite, showing instead an inhibition of the Th2 response and an induction of Th1 activity (43–46). We postulate that these diverse outcomes, in part, stem from effects on the synthesis of lipid mediators downstream of COX2 other than PGE2, such as PGD_2_ and PGI_2_, which have been shown to exert diverse immunomodulatory effects, that include modulation of Th1 and Th2 differentiation (47–54).

These *in vitro* findings were largely recapitulated *in vivo*, as targeted PGE2 inhibition reduced the Th2 response following immunization with *S. mansoni* eggs. We found that there was lower Th2 cell expansion in immunized mice treated with the inhibitor as well as reduced Th2 cell cytokine production following antigen-specific restimulation. It is worth noting that egg-induced Th1- and Th17-associated cytokine production was also reduced, suggesting a more general dampening effect of mPGES inhibition on *S. mansoni* egg-driven Th cell priming that is not limited to the Th2 response specifically. It is known that PGE2 signaling can contribute to DC maturation, antigen uptake, and migration to lymph nodes (55–57), and as such, additional studies would be required to evaluate to what extent the effects of PGE2 inhibition on T cell priming, in the context of *S. mansoni* egg challenge, are secondary to changes in DC biology as a whole.

In conclusion, we show here that SEA is able to directly bind to Dectin-1 and Dectin-2 on moDCs, the latter in a partially high-mannose-dependent manner. We also reported on the importance of high-mannose residues present in SEA in inducing the previously identified PGE2/OX40L signaling axis (10) that licenses DCs to promote a Th2 immune response. Additionally, we found that the specific targeting of PGE2 synthesis, by chemical inhibition of mPGES, is able to impair Th2 priming by DCs both *in vitro* and *in vivo*. As the expression of high-mannose glycans is shared with several other parasitic helminths, such as *Fasciola hepatica* (58) and *Brugia malayi* (23), it will be interesting, in future studies, to explore whether high-mannose glycan-driven Dectin-2/PGE2/OX40L signaling axis is a more common pathway through which helminths elicit Th2 responses. Finally, our data provide first proof of principle that targeting PGE2 synthesis with specific chemical inhibitors could be a strategy to dampen pathological type 2 immunity in the context of schistosomiasis and possibly also in other type-2 immunity driven diseases, such as allergies.

## Data availability statement

The raw data supporting the conclusions of this article will be made available by the authors, without undue reservation.

## Ethics statement

The studies involving humans were approved by Sanquin National Blood donation Bank. The studies were conducted in accordance with the local legislation and institutional requirements. The human samples used in this study were acquired from voluntary blood donations to Sanquin Blood bank. Written informed consent for participation was not required from the participants or the participants’ legal guardians/next of kin in accordance with the national legislation and institutional requirements. The animal study was approved by Dutch Central Authority for Scientific Procedures on Animals (CCD). The study was conducted in accordance with the local legislation and institutional requirements.

## Author contributions

LA: Formal analysis, Investigation, Methodology, Visualization, Writing – original draft. RvR: Formal analysis, Investigation, Methodology, Writing – review & editing. TP: Investigation, Methodology, Writing – review & editing. FO: Investigation, Writing – review & editing. TV: Formal analysis, Investigation, Methodology, Writing – review & editing. MGh: Data curation, Formal analysis, Investigation, Methodology, Writing – review & editing. AvD: Formal analysis, Supervision, Writing – review & editing. GS: Resources, Writing – review & editing. JL: Resources, Writing – review & editing. HI: Resources, Writing – review & editing. MK: Resources, Writing – review & editing. P-JJ: Resources, Supervision, Writing – review & editing. MGi: Resources, Supervision, Writing – review & editing. CH: Conceptualization, Supervision, Writing – review & editing. BE: Conceptualization, Funding acquisition, Project administration, Supervision, Writing – original draft.

## Supplementary material

Supplementary Figure 1 MALDI-TOF MS of Mock- and EndoH-treated soluble egg antigen N-glycans. MALDI-TOF mass spectra of PNGase-A released AA-labeled N-glycans of Mock-treated (top) or EndoH (bottom)-treated SEA.

Supplementary Figure 2 Purification of Man9 from human serum and AEAB labeling. **(A)** RP-HPLC profile of high-mannose glycans cleaved from human serum with EndoH and **(B)** MALDI-TOF mass spectrum of Man9 after purification as described in “Materials and methods”. **(C)** MALDI-TOF mass spectra of AEAB-labeled Man9 (top) and AA-labeled Man9 (bottom). Successful AEAB labeling, as described in “Materials and methods”, can be identified by an increase of 42 m/z ratio of the peaks, with Man9-AEAB having a m/z ratio of 1,843.674 (top) and Man9-AA a m/z ratio of 1,801.594 (bottom). **(D)** RP-HPLC profile of Man9-AEAB generated and purified as described in “Materials and methods”.

Supplementary Figure 3 Nanoparticle Dectin binding ELISA. Dectin-1 **(A)** and Dectin-2 **(B)** binding ELISA to Man9-coated nanoparticles (Man9-NP) or inactivated nanoparticles (øNP). Data are from one experiment.

Supplementary Figure 4 Effects of COX and mPGES inhibitor on lipid species synthesis. **(A)** PGF2a, PGE2, and TxB2 concentrations in supernatants from moDC cultures after stimulation with the indicated reagents. The cells were stimulated with Zymosan and lipopolysaccharide to induce high levels of COX-dependent lipid species, along with either CIII to inhibit mPGES or indomethacin to inhibit COX. To confirm the specificity of CIII for the inhibition of PGE2 synthesis, we measured not only PGE2 levels but also mPGES-independent, COX-dependent lipids, such as PGF2a and TxB2, and compared the effect of CIII to indomethacin. Data points represent data from two individual donors.

## References

[1] Kapsenberg ML. Dendritic-cell control of pathogen-driven T-cell polarization. Nat Rev Immunol. (2003) 3:984–93. doi: 10.1038/nri1246 14647480

[2] Cabeza-Cabrerizo M Cardoso A Minutti CM Pereira da Costa M Reis e Sousa C. Dendritic cells revisited. Annu Rev Immunol. (2021) 39:131–66. doi: 10.1146/annurev-immunol-061020-053707 33481643

[3] MacDonald AS Straw AD Bauman B Pearce EJ. CD8– dendritic cell activation status plays an integral role in influencing th2 response development1. J Immunol. (2001) 167:1982–8. doi: 10.4049/jimmunol.167.4.1982 11489979

[4] Harris NL Loke P. Recent advances in type-2-cell-mediated immunity: insights from helminth infection. Immunity. (2017) 47:1024–36. doi: 10.1016/j.immuni.2017.11.015 29262347

[5] Everts B Perona-Wright G Smits HH Hokke CH van der Ham AJ Fitzsimmons CM. Omega-1, a glycoprotein secreted by Schistosoma mansoni eggs, drives Th2 responses. J Exp Med. (2009) 206:1673–80. doi: 10.1084/jem.20082460 PMC2722183 19635864

[6] Steinfelder S Andersen JF Cannons JL Feng CG Joshi M Dwyer D. The major component in schistosome eggs responsible for conditioning dendritic cells for Th2 polarization is a T2 ribonuclease (omega-1). J Exp Med. (2009) 206:1681–90. doi: 10.1084/jem.20082462 PMC2722182 19635859

[7] Phythian-Adams AT Cook PC Lundie RJ Jones LH Smith KA Barr TA. CD11c depletion severely disrupts Th2 induction and development in vivo. J Exp Med. (2010) 207:2089–96. doi: 10.1084/jem.20100734 PMC2947067 20819926

[8] Hokke CH Yazdanbakhsh M. Schistosome glycans and innate immunity. Parasite Immunol. (2005) 27:257–64. doi: 10.1111/j.1365-3024.2005.00781.x 16138846

[9] Everts B Hussaarts L Driessen NN Meevissen MHJ Schramm G van der Ham AJ. Schistosome-derived omega-1 drives Th2 polarization by suppressing protein synthesis following internalization by the mannose receptor. J Exp Med. (2012) 209:1753–67, S1. doi: 10.1084/jem.20111381 22966004 PMC3457738

[10] Kaisar MMM Ritter M Del Fresno C Jónasdóttir HS van der Ham AJ Pelgrom LR. Dectin-1/2-induced autocrine PGE2 signaling licenses dendritic cells to prime Th2 responses. PloS Biol. (2018) 16:e2005504. doi: 10.1371/journal.pbio.2005504 29668708 PMC5927467

[11] Mickum ML Prasanphanich NS Song X Dorabawila N Mandalasi M Lasanajak Y. Identification of antigenic glycans from schistosoma mansoni by using a shotgun egg glycan microarray. Infect Immun. (2016) 84:1371–86. doi: 10.1128/IAI.01349-15 PMC4862720 26883596

[12] Okano M Satoskar AR Nishizaki K Abe M Harn DA. Induction of Th2 responses and IgE is largely due to carbohydrates functioning as adjuvants on Schistosoma mansoni egg antigens. J Immunol Baltim Md 1950. (1999) 163:6712–7. doi: 10.4049/jimmunol.163.12.6712 10586068

[13] van Vliet SJ van Liempt E Saeland E Aarnoudse CA Appelmelk B Irimura T. Carbohydrate profiling reveals a distinctive role for the C-type lectin MGL in the recognition of helminth parasites and tumor antigens by dendritic cells. Int Immunol. (2005) 17:661–9. doi: 10.1093/intimm/dxh246 15802303

[14] Meevissen MHJ Driessen NN Smits HH Versteegh R van Vliet SJ van Kooyk Y. Specific glycan elements determine differential binding of individual egg glycoproteins of the human parasite Schistosoma mansoni by host C-type lectin receptors. Int J Parasitol. (2012) 42:269–77. doi: 10.1016/j.ijpara.2012.01.004 22673410

[15] van Liempt E van Vliet SJ Engering A García Vallejo JJ Bank CMC Sanchez-Hernandez M. Schistosoma mansoni soluble egg antigens are internalized by human dendritic cells through multiple C-type lectins and suppress TLR-induced dendritic cell activation. Mol Immunol. (2007) 44:2605–15. doi: 10.1016/j.molimm.2006.12.012 17241663

[16] Gringhuis SI den Dunnen J Litjens M van der Vlist M Geijtenbeek TBH. Carbohydrate-specific signaling through the DC-SIGN signalosome tailors immunity to Mycobacterium tuberculosis, HIV-1 and Helicobacter pylori. Nat Immunol. (2009) 10:1081–8. doi: 10.1038/ni.1778 19718030

[17] Gringhuis SI Kaptein TM Wevers BA Mesman AW Geijtenbeek TBH. Fucose-specific DC-SIGN signalling directs T helper cell type-2 responses via IKKϵ- and CYLD-dependent Bcl3 activation. Nat Commun. (2014) 5:3898. doi: 10.1038/ncomms4898 24867235

[18] van Die I van Vliet SJ Nyame AK Cummings RD Bank CMC Appelmelk B. The dendritic cell-specific C-type lectin DC-SIGN is a receptor for Schistosoma mansoni egg antigens and recognizes the glycan antigen Lewis x. Glycobiology. (2003) 13:471–8. doi: 10.1093/glycob/cwg052 12626400

[19] Brown GD Gordon S. Immune recognition. A new receptor for beta-glucans. Nature. (2001) 413:36–7. doi: 10.1038/35092620 11544516

[20] McGreal EP Rosas M Brown GD Zamze S Wong SYC Gordon S. The carbohydrate-recognition domain of Dectin-2 is a C-type lectin with specificity for high mannose. Glycobiology. (2006) 16:422–30. doi: 10.1093/glycob/cwj077 16423983

[21] Smit CH van Diepen A Nguyen DL Wuhrer M Hoffmann KF Deelder AM. Glycomic analysis of life stages of the human parasite schistosoma mansoni reveals developmental expression profiles of functional and antigenic glycan motifs. Mol Cell Proteomics MCP. (2015) 14:1750–69. doi: 10.1074/mcp.M115.048280 PMC4587318 25883177

[22] Dunne DW Agnew AM Modha J Doenhoff MJ. Schistosoma mansoni egg antigens: preparation of rabbit antisera with monospecific immunoprecipitating activity, and their use in antigen characterization. Parasite Immunol. (1986) 8:575–86. doi: 10.1111/j.1365-3024.1986.tb00871.x 3101031

[23] Petralia LMC van Diepen A Lokker LA Nguyen DL Sartono E Khatri V. Mass spectrometric and glycan microarray-based characterization of the filarial nematode brugia malayi glycome reveals anionic and zwitterionic glycan antigens. Mol Cell Proteomics MCP. (2022) 21:100201. doi: 10.1016/j.mcpro.2022.100201 35065273 PMC9046957

[24] Zhu Y Liu X Zhang Y Wang Z Lasanajak Y Song X. Anthranilic acid as a versatile fluorescent tag and linker for functional glycomics. Bioconjug Chem. (2018) 29:3847–55. doi: 10.1021/acs.bioconjchem.8b00678 PMC6309456 30380836

[25] Hussaarts L Smits HH Schramm G van der Ham AJ van der Zon GC Haas H. Rapamycin and omega-1: mTOR-dependent and -independent Th2 skewing by human dendritic cells. Immunol Cell Biol. (2013) 91:486–9. doi: 10.1038/icb.2013.31 23835553

[26] Giera M Ioan-Facsinay A Toes R Gao F Dalli J Deelder AM. Lipid and lipid mediator profiling of human synovial fluid in rheumatoid arthritis patients by means of LC-MS/MS. Biochim Biophys Acta. (2012) 1821:1415–24. doi: 10.1016/j.bbalip.2012.07.011 PMC3433634 22841830

[27] Decout A Silva-Gomes S Drocourt D Blattes E Rivière M Prandi J. Deciphering the molecular basis of mycobacteria and lipoglycan recognition by the C-type lectin Dectin-2. Sci Rep. (2018) 8:16840. doi: 10.1038/s41598-018-35393-5 30443026 PMC6237770

[28] Bergqvist F Ossipova E Idborg H Raouf J Checa A Englund K. Inhibition of mPGES-1 or COX-2 results in different proteomic and lipidomic profiles in A549 lung cancer cells. Front Pharmacol. (2019) 10:636. doi: 10.3389/fphar.2019.00636 31231223 PMC6567928

[29] Kitai Y Sato K Tanno D Yuan X Umeki A Kasamatsu J. Role of dectin-2 in the phagocytosis of cryptococcus neoformans by dendritic cells. Infect Immun. (2021) 89:e0033021. doi: 10.1128/IAI.00330-21 34251289 PMC8445189

[30] Tanno D Yokoyama R Kawamura K Kitai Y Yuan X Ishii K. Dectin-2-mediated signaling triggered by the cell wall polysaccharides of Cryptococcus neoformans. Microbiol Immunol. (2019) 63:500–12. doi: 10.1111/1348-0421.12746 31544981

[31] Saijo S Iwakura Y. Dectin-1 and Dectin-2 in innate immunity against fungi. Int Immunol. (2011) 23:467–72. doi: 10.1093/intimm/dxr046 21677049

[32] Guasconi L Burstein VL Beccacece I Mena C Chiapello LS Masih DT. Dectin-1 on macrophages modulates the immune response to Fasciola hepatica products through the ERK signaling pathway. Immunobiology. (2018) 223:834–8. doi: 10.1016/j.imbio.2018.08.004 30197196

[33] de los Reyes Jiménez M Lechner A Alessandrini F Bohnacker S Schindela S Trompette A. An anti-inflammatory eicosanoid switch mediates the suppression of type-2 inflammation by helminth larval products. Sci Transl Med. (2020) 12:eaay0605. doi: 10.1126/scitranslmed.aay0605 32321863

[34] Saijo S Fujikado N Furuta T hyun CS Kotaki H Seki K. Dectin-1 is required for host defense against Pneumocystis carinii but not against Candida albicans. Nat Immunol. (2007) 8:39–46. doi: 10.1038/ni1425 17159982

[35] Chiba S Ikushima H Ueki H Yanai H Kimura Y Hangai S. Recognition of tumor cells by Dectin-1 orchestrates innate immune cells for anti-tumor responses. eLife. (2014) 3:e04177. doi: 10.7554/eLife.04177 25149452 PMC4161974

[36] Ariizumi K Shen GL Shikano S Xu S Ritter R Kumamoto T. Identification of a novel, dendritic cell-associated molecule, dectin-1, by subtractive cDNA cloning. J Biol Chem. (2000) 275:20157–67. doi: 10.1074/jbc.M909512199 10779524

[37] Hinterwirth H Wiedmer SK Moilanen M Lehner A Allmaier G Waitz T. Comparative method evaluation for size and size-distribution analysis of gold nanoparticles. J Sep Sci. (2013) 36:2952–61. doi: 10.1002/jssc.201300460 23857600

[38] Tobola F Wiltschi B. One, two, many: Strategies to alter the number of carbohydrate binding sites of lectins. Biotechnol Adv. (2022) 60:108020. doi: 10.1016/j.biotechadv.2022.108020 35868512

[39] Kaliński P Schuitemaker JH Hilkens CM Kapsenberg ML. Prostaglandin E2 induces the final maturation of IL-12-deficient CD1a+CD83+ dendritic cells: the levels of IL-12 are determined during the final dendritic cell maturation and are resistant to further modulation. J Immunol Baltim Md 1950. (1998) 161:2804–9. doi: 10.4049/jimmunol.161.6.2804 9743339

[40] Meyer F Ramanujam KS Gobert AP James SP Wilson KT. Cutting edge: cyclooxygenase-2 activation suppresses Th1 polarization in response to Helicobacter pylori. J Immunol Baltim Md 1950. (2003) 171:3913–7. doi: 10.4049/jimmunol.171.8.3913 14530307

[41] Teloni R Giannoni F Rossi P Nisini R Gagliardi MC. Interleukin-4 inhibits cyclo-oxygenase-2 expression and prostaglandin E2 production by human mature dendritic cells. Immunology. (2007) 120:83–9. doi: 10.1111/j.1365-2567.2006.02482.x PMC2265872 17059508

[42] Markosyan N Chen EP Ndong VN Yao Y Sterner CJ Chodosh LA. Deletion of cyclooxygenase 2 in mouse mammary epithelial cells delays breast cancer onset through augmentation of type 1 immune responses in tumors. Carcinogenesis. (2011) 32:1441–9. doi: 10.1093/carcin/bgr134 PMC3975167 21771729

[43] Jaffar Z Wan KS Roberts K. A key role for prostaglandin I2 in limiting lung mucosal Th2, but not Th1, responses to inhaled allergen. J Immunol Baltim Md 1950. (2002) 169:5997–6004. doi: 10.4049/jimmunol.169.10.5997 12421986

[44] Carey MA Germolec DR Bradbury JA Gooch RA Moorman MP Flake GP. Accentuated T helper type 2 airway response after allergen challenge in cyclooxygenase-1-/- but not cyclooxygenase-2-/- mice. Am J Respir Crit Care Med. (2003) 167:1509–15. doi: 10.1164/rccm.200211-1383OC 12626351

[45] Nagamachi M Sakata D Kabashima K Furuyashiki T Murata T Segi-Nishida E. Facilitation of Th1-mediated immune response by prostaglandin E receptor EP1. J Exp Med. (2007) 204:2865–74. doi: 10.1084/jem.20070773 PMC2118516 17967902

[46] Hamada T Tsuchihashi S Avanesyan A Duarte S Moore C Busuttil RW. Cyclooxygenase-2 deficiency enhances Th2 immune responses and impairs neutrophil recruitment in hepatic ischemia/reperfusion injury. J Immunol Baltim Md 1950. (2008) 180:1843–53. doi: 10.4049/jimmunol.180.3.1843 PMC3589995 18209082

[47] Steinbrink K Paragnik L Jonuleit H Tüting T Knop J Enk AH. Induction of dendritic cell maturation and modulation of dendritic cell-induced immune responses by prostaglandins. Arch Dermatol Res. (2000) 292:437–45. doi: 10.1007/s004030000159 11000287

[48] Tanaka K Ogawa K Sugamura K Nakamura M Takano S Nagata K. Cutting edge: differential production of prostaglandin D2 by human helper T cell subsets. J Immunol Baltim Md 1950. (2000) 164:2277–80. doi: 10.4049/jimmunol.164.5.2277 10679060

[49] Hirai H Tanaka K Yoshie O Ogawa K Kenmotsu K Takamori Y. Prostaglandin D2 selectively induces chemotaxis in T helper type 2 cells, eosinophils, and basophils via seven-transmembrane receptor CRTH2. J Exp Med. (2001) 193:255–61. doi: 10.1084/jem.193.2.255 PMC2193345 11208866

[50] Takahashi Y Tokuoka S Masuda T Hirano Y Nagao M Tanaka H. Augmentation of allergic inflammation in prostanoid IP receptor deficient mice. Br J Pharmacol. (2002) 137:315–22. doi: 10.1038/sj.bjp.0704872 PMC1573495 12237250

[51] Nagao K Tanaka H Komai M Masuda T Narumiya S Nagai H. Role of prostaglandin I2 in airway remodeling induced by repeated allergen challenge in mice. Am J Respir Cell Mol Biol. (2003) 29:314–20. doi: 10.1165/rcmb.2003-0035OC 12676807

[52] Mandal AK Zhang Z Ray R Choi MS Chowdhury B Pattabiraman N. Uteroglobin represses allergen-induced inflammatory response by blocking PGD2 receptor-mediated functions. J Exp Med. (2004) 199:1317–30. doi: 10.1084/jem.20031666 PMC2211805 15148333

[53] Schuligoi R Sedej M Waldhoer M Vukoja A Sturm EM Lippe IT. Prostaglandin H2 induces the migration of human eosinophils through the chemoattractant receptor homologous molecule of Th2 cells, CRTH2. J Leukoc Biol. (2009) 85:136–45. doi: 10.1189/jlb.0608387 18835884

[54] Nakajima S Honda T Sakata D Egawa G Tanizaki H Otsuka A. Prostaglandin I2-IP signaling promotes Th1 differentiation in a mouse model of contact hypersensitivity. J Immunol Baltim Md 1950. (2010) 184:5595–603. doi: 10.4049/jimmunol.0903260 20400695

[55] Legler DF Krause P Scandella E Singer E Groettrup M. Prostaglandin E2 is generally required for human dendritic cell migration and exerts its effect via EP2 and EP4 receptors. J Immunol Baltim Md 1950. (2006) 176:966–73. doi: 10.4049/jimmunol.176.2.966 16393982

[56] van Helden SFG Krooshoop DJEB Broers KCM Raymakers RAP Figdor CG van Leeuwen FN. A critical role for prostaglandin E2 in podosome dissolution and induction of high-speed migration during dendritic cell maturation. J Immunol Baltim Md 1950. (2006) 177:1567–74. doi: 10.4049/jimmunol.177.3.1567 16849464

[57] Krause P Bruckner M Uermösi C Singer E Groettrup M Legler DF. Prostaglandin E(2) enhances T-cell proliferation by inducing the costimulatory molecules OX40L, CD70, and 4-1BBL on dendritic cells. Blood. (2009) 113:2451–60. doi: 10.1182/blood-2008-05-157123 19029446

[58] Ravidà A Aldridge AM Driessen NN Heus FAH Hokke CH O’Neill SM. Fasciola hepatica surface coat glycoproteins contain mannosylated and phosphorylated N-glycans and exhibit immune modulatory properties independent of the mannose receptor. PloS Negl Trop Dis. (2016) 10:e0004601. doi: 10.1371/journal.pntd.0004601 27104959 PMC4841591

