# Supplementary figures and images for: High-mannose glycans from Schistosoma mansoni eggs are important for priming of Th2 responses via Dectin-2 and prostaglandin E2
Source: Front Immunol. 2024 Apr 29;15:1372927. doi: 10.3389/fimmu.2024.1372927 (PMC11089121; doi:10.3389/fimmu.2024.1372927)

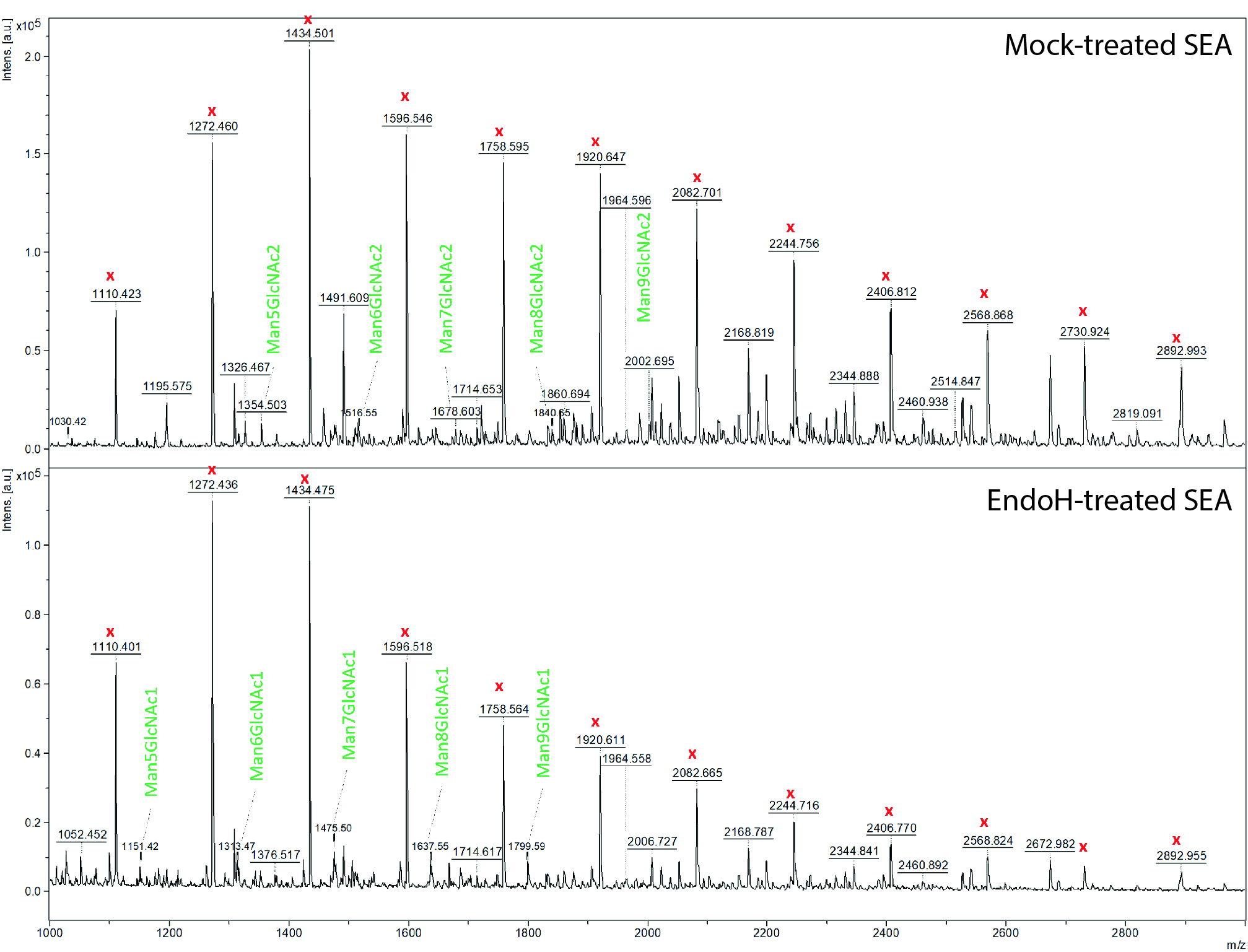

Supplement: Supplementary Figure 1 — MALDI-TOF MS of Mock- and EndoH-treated soluble egg antigen N-glycans. MALDI-TOF mass spectra of PNGase-A released AA-labeled N-glycans of Mock-treated (top) or EndoH (bottom)-treated SEA. [file Image_1.jpeg]

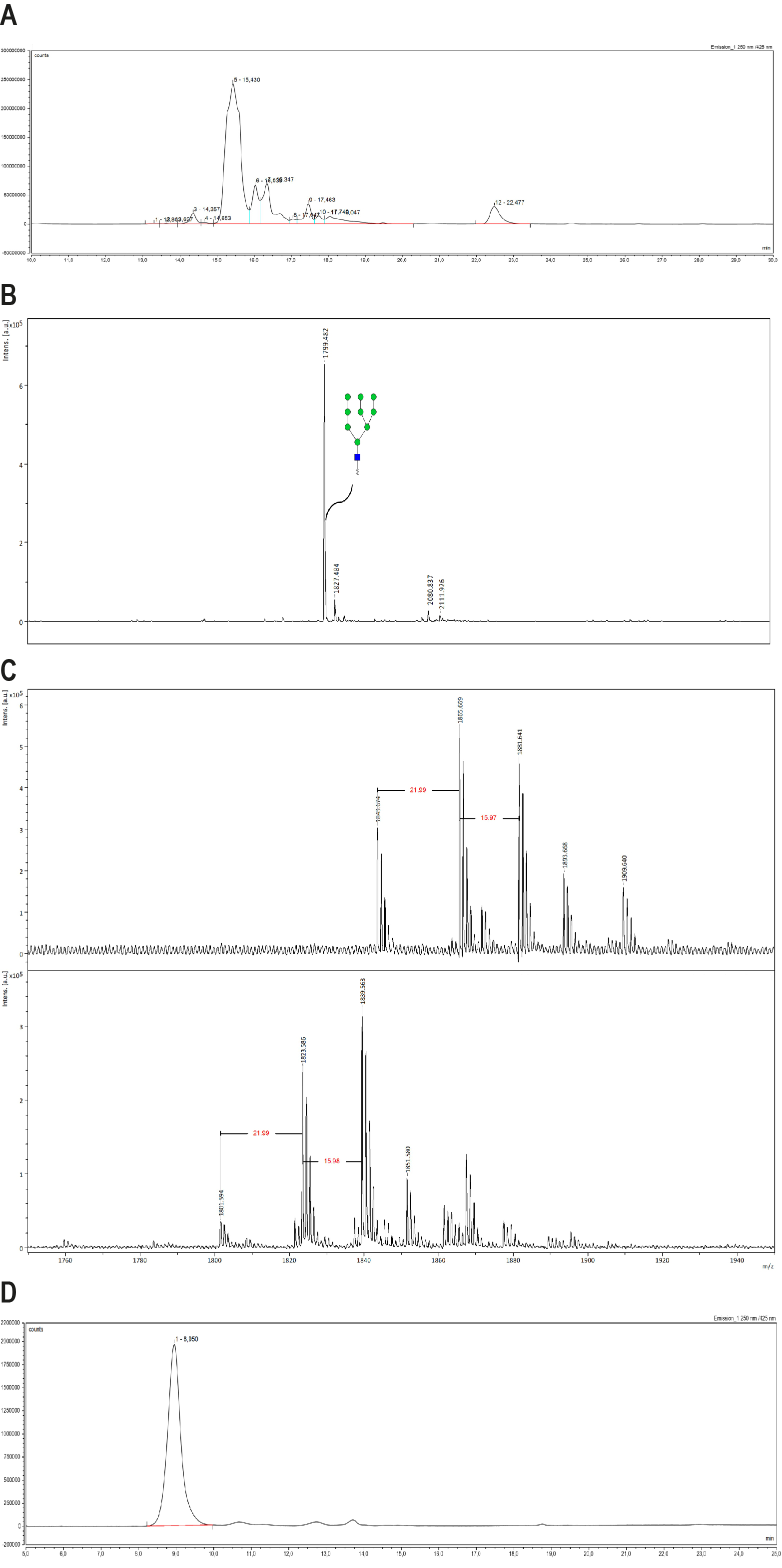

Supplement: Supplementary Figure 2 — Purification of Man9 from human serum and AEAB labeling. (A) RP-HPLC profile of high-mannose glycans cleaved from human serum with EndoH and (B) MALDI-TOF mass spectrum of Man9 after purification as described in “Materials and methods”. (C) MALDI-TOF mass spectra of AEAB-labeled Man9 (top) and AA-labeled Man9 (bottom). Successful AEAB labeling, as described in “Materials and methods”, can be identified by an increase of 42 m/z ratio of the peaks, with Man9-AEAB having a m/z ratio of 1,843.674 (top) and Man9-AA a m/z ratio of 1,801.594 (bottom). (D) RP-HPLC profile of Man9-AEAB generated and purified as described in “Materials and methods”. [file Image_2.jpeg]

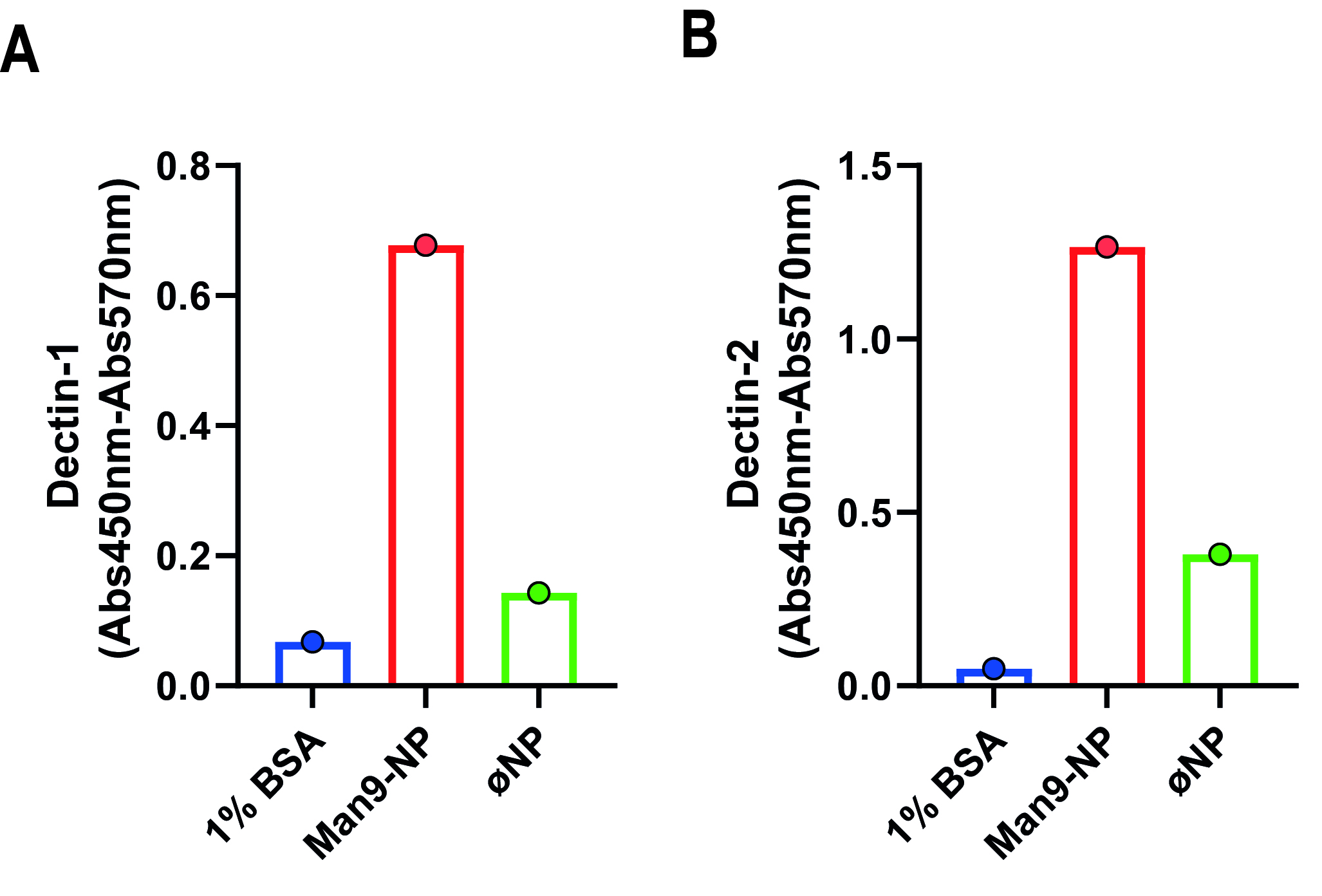

Supplement: Supplementary Figure 3 — Nanoparticle Dectin binding ELISA. Dectin-1 (A) and Dectin-2 (B) binding ELISA to Man9-coated nanoparticles (Man9-NP) or inactivated nanoparticles (øNP). Data are from one experiment. [file Image_3.jpeg]

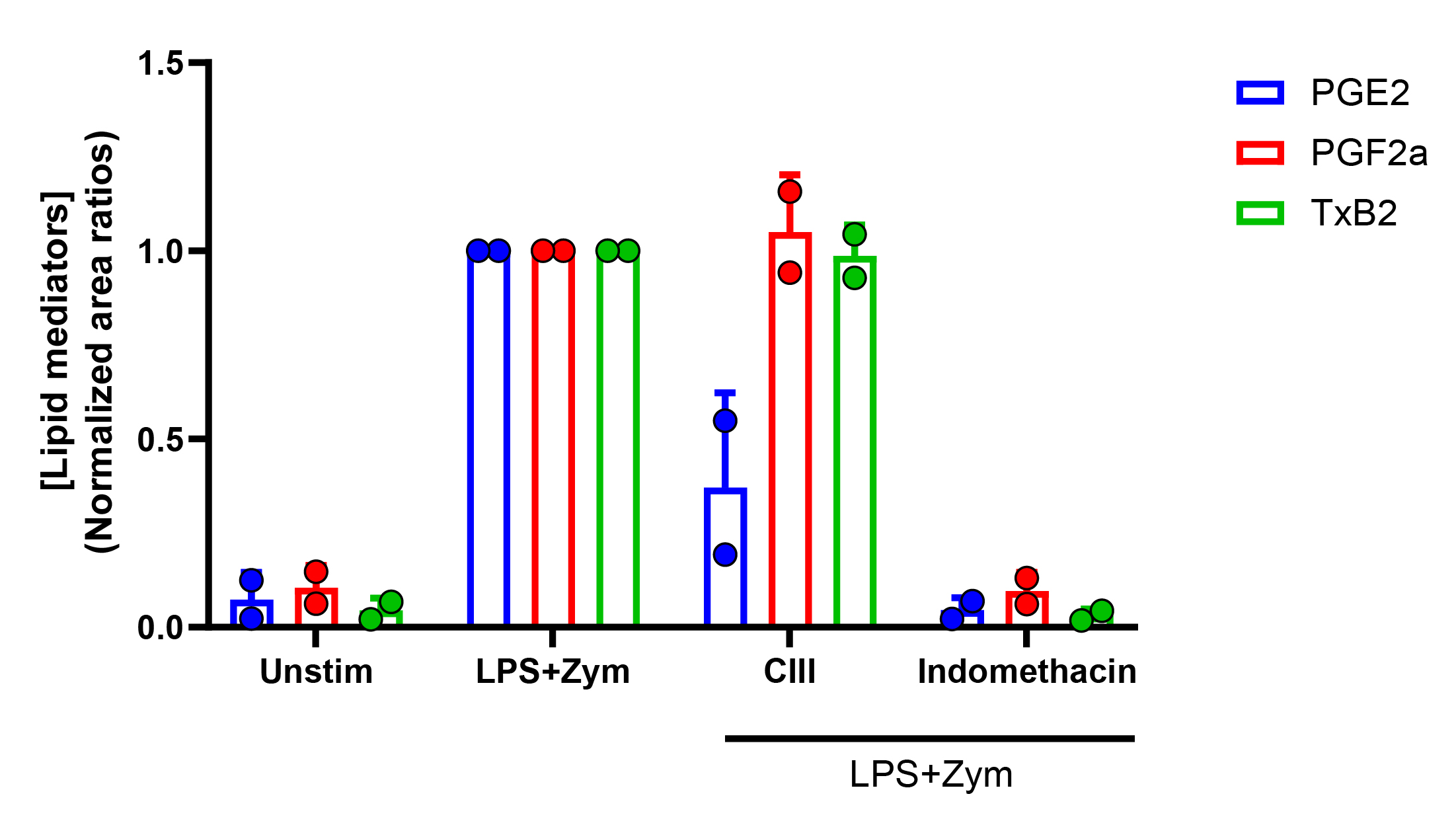

Supplement: Supplementary Figure 4 — Effects of COX and mPGES inhibitor on lipid species synthesis. (A) PGF2a, PGE2, and TxB2 concentrations in supernatants from moDC cultures after stimulation with the indicated reagents. The cells were stimulated with Zymosan and lipopolysaccharide to induce high levels of COX-dependent lipid species, along with either CIII to inhibit mPGES or indomethacin to inhibit COX. To confirm the specificity of CIII for the inhibition of PGE2 synthesis, we measured not only PGE2 levels but also mPGES-independent, COX-dependent lipids, such as PGF2a and TxB2, and compared the effect of CIII to indomethacin. Data points represent data from two individual donors. [file Image_4.jpeg]
